# Innovative sterile male release strategies for *Aedes* mosquito control: progress and challenges in integrating evidence of mosquito population suppression with epidemiological impact

**DOI:** 10.1186/s40249-024-01258-5

**Published:** 2024-12-03

**Authors:** Arya Rahul, Appadurai Daniel Reegan, A. N. Shriram, Florence Fouque, Manju Rahi

**Affiliations:** 1 ICMR-Vector Control Research Centre, Indira Nagar, Puducherry, India; 2 Academy of Scientific and Innovation Research (AcSIR), CSIR-Human Resource Development Centre, (CSIR-HRDC) Campus, Ghaziabad, Uttar Pradesh India; 3 World Health Organization, TDR, Geneva, Switzerland

**Keywords:** Sterile insect technique, Incompatible insect technique, Trial, *Aedes*, Review

## Abstract

**Background:**

*Aedes* mosquitoes pose a significant global threat as vectors for several debilitating arboviruses, including dengue, Zika, yellow fever, and chikungunya. Their unique breeding habits, behavior, and daytime activity complicate control efforts, prompting the search for innovative solutions. The sterile insect technique (SIT) and incompatible insect technique (IIT) are promising new techniques under investigation. This review synthesizes findings from field trials on SIT and/or IIT for *Aedes* mosquito control.

**Methods:**

A scoping review was conducted through comprehensive searches on Scopus, Web of Science, MEDLINE, PubMed, and preprint repositories up to April 25, 2024. Studies were initially screened for relevance based on their titles and abstracts, followed by a full-text review conducted by two independent extractors. Only field trials with control groups were included, with the final assessment focusing on trials reporting epidemiological outcomes. Data were abstracted into templates, emphasizing study design, intervention details, and outcomes. The review adhered to the PRISMA-ScR guidelines.

**Results:**

The search identified 21 field trials in various countries against *Aedes* mosquitoes. These trials employed diverse methodologies and mosquito release strategies, achieving varying levels of mosquito population suppression. Notably, two SIT and two *Wolbachia*-based IIT trials reported epidemiological outcomes, including reductions in dengue incidence and associated risk ratios. However, the reliance on national surveillance data for assessing dengue incidence suggests caution due to the potential underreporting of subclinical cases.

**Conclusions:**

The review underscores the promise of SIT and IIT for controlling *Aedes* mosquito populations, citing successful reductions in mosquito densities and dengue transmission. However, it calls for more rigorous study designs and standardized methodologies, as well as the adoption of comprehensive frameworks to accurately assess the effectiveness of these interventions. Future research should focus on bridging gaps in real-world effectiveness by addressing factors such as feasibility, acceptability, scalability, and cost, which are crucial for guiding their successful large-scale deployment in any country.

**Supplementary Information:**

The online version contains supplementary material available at 10.1186/s40249-024-01258-5.

## Background

*Aedes* mosquitoes are notorious vectors responsible for transmitting a myriad debilitating arboviruses, including dengue virus, Zika virus (ZIKV), yellow fever virus, and chikungunya virus (CHIKV). These diseases inflict a heavy toll on human health, causing widespread morbidity and mortality in affected regions across the globe. The intricate dynamics of *Aedes*-borne diseases demand comprehensive and targeted control measures to mitigate their impact effectively. Dengue fever stands as a burgeoning threat among arboviral diseases considering its endemicity in 128 countries compared to only 9 countries in the 1970s [1]. With the World Health Organization (WHO) reporting a staggering rise in cases globally, surpassing 5 million in 2019 alone, with an estimated clinical manifestation in 96 million people each year, the urgency to curtail this disease has never been more acute [1, 2]. There were reports of more than six million dengue cases and over 6000 deaths linked to dengue from 92 countries and territories in 2023 [3]. A global surge in dengue cases and deaths and expansion to new areas has prompted a health emergency appeal by WHO in 2024 [4]. The lack of commercially available effective antivirals and the potential limitations of dengue vaccines underscores the critical importance of disease prevention, with a primary emphasis on vector control [2]. Epidemics caused by emerging and re-emerging *Aedes*-borne arboviral infections like chikungunya, Zika, and yellow fever also hold concerns, especially in the rapidly urbanizing world [5]. Published data from 2010 to 2019 suggests that the ZIKV and CHIKV contributed to an average annual loss of over 106,000 lives and an estimated 44,000 disability-adjusted life years (DALYs) [6].

*Aedes* mosquitoes possess optimal adaptations for viral transmission including anthropophilic behavior, human-centric habitats like small containers, daytime feeding, and multiple host-seeking behaviors. Urbanization and climate change further exacerbate this by expanding suitable breeding habitats and creating conducive environmental conditions for vector to thrive [7]. Despite concerted efforts, the arsenal against *Aedes* mosquitoes remains constrained. Chemical larvicides and adulticides, though effective to some extent, remain subjects of ongoing debate concerning their impact on human health and the environment [8, 9]. Source reduction techniques, though promising, encounter practical constraints. One of the major impediments is the essential role of multiple stakeholders ranging from the health department to the urban development department and civic bodies. It also becomes more difficult to deploy particularly in water-scarce regions. Biological control methods like larvicide *Bacillus thuringiensis israelensis*, larvivorous fish, and other natural predators offer a glimpse of hope but lack robust epidemiological evidence for sustained dengue control [10]. In the face of these limitations, the quest for innovative and sustainable solutions is intensifying. Newer techniques for mosquito control like genetically modified mosquitoes, irradiated mosquitoes, and *Wolbachia*-transfected mosquitoes are being explored to manage mosquito vectors.

Among the strategies and tools currently under investigation, the sterile insect technique (SIT) stands out with a proven track record in controlling agricultural pests like fruit flies, screwworms, moths, and tsetse fly [11]. SIT operates as a method of biological mosquito control, wherein sterile male insects are released into the field to mate with native females of the same species, gradually reducing the population's reproductive potential over time [12]. In the context of vector-borne diseases affecting humans, this technique involves sterilizing male mosquitoes in the laboratory using irradiation/chemosterilants/genetic modification techniques before releasing them into the wild, resulting in inseminations that fail to produce offspring [13]. Several countries are exploring SIT for *Aedes* mosquito control due to its environmentally friendly and species-specific effectiveness against target mosquitoes. In parallel, the incompatible insect technique (IIT) presents another biological control method, deploying sterile male insects containing *Wolbachia* to disrupt the reproductive cycle of native females [14]. By preventing the hatching of eggs, IIT effectively suppresses population growth, serving as a barrier to *Aedes* mosquito reproduction. In recent years, numerous field trials using SIT against *Aedes* mosquitoes have been conducted globally, with few also incorporating the IIT. Reports have indicated the combined efficacy of SIT with IIT against *Ae. albopictus* mosquitoes, showcasing their potential synergy in population suppression efforts [15]. To provide comprehensive guidance for pilot evaluations, scale-up, and operational implementation of SIT, the WHO has developed a ‘Guidance Framework for Testing the Sterile Insect Technique as a Vector Control Tool against *Aedes*-Borne Diseases’ [16]. Additionally, the guidance document by the Vector Control Advisory Group outlines standardized methodologies for designing Phase III vector control field trials to evaluate the efficacy of novel interventions [17]. The effectiveness of the intervention is usually measured through entomological outcomes and epidemiological impacts. Understanding the epidemiological outcomes is critical to both evaluate the effectiveness of such interventions in reducing *Aedes*-borne disease transmission and to guide their integration into vector control programs. Therefore, this review aims to explore and synthesize findings from SIT and IIT trials for *Aedes* mosquito control, with a focus on epidemiological outcomes, study methodologies, the scalability and feasibility of the intervention, and research gaps. By doing so, this review will provide insights into the potential of SIT and IIT as sustainable vector control strategies, as well as inform the design and implementation of future trials and policy decisions on vector control strategies.

## Methods

A scoping review approach was chosen to comprehensively map the diverse and expansive research on SIT for *Aedes* mosquito control. This methodology allows for a broad overview of existing evidence, highlights key advancements, and identifies research gaps, thereby providing a foundation for future studies.

### Search strategy

A scoping review was undertaken through comprehensive searches conducted on Scopus, Web of Science, MEDLINE, and PubMed databases. Additionally, an opportunistic search of preprint repositories was undertaken to augment the dataset. The initial searches were executed in January 2024, and a supplementary search was performed on August 20, 2024, to ensure comprehensive inclusion of all relevant literature published up to that date. A subsequent search of the bibliographies of the selected articles was also done by a snowballing method. The methods followed the guidelines outlined in the Preferred Reporting Items for Systematic Reviews and Meta-Analyses Extension for Scoping Reviews (PRISMA-ScR) statement [18]. The search strategy employed the following terms: (“Sterile Insect Technique”) OR (“Incompatible Insect Technique”) AND (“Field trial” OR Effectiveness) AND (Dengue OR Chikungunya OR Zika OR *Aedes*). This controlled vocabulary search was supplemented by a comprehensive search using synonyms found in the literature. The detailed search strategy for each database is given in Additional file 1. A thorough screening process was employed to avoid duplication, examining each result by author, title, journal, and publication date. Subsequently, the relevance of the studies was analyzed based solely on the title and abstract. For studies deemed relevant, the full text was meticulously reviewed by two independent data extractors for final evaluation. Any discrepancies were resolved through consensus via discussion when required. During the screening and data abstraction process, bibliographies of pertinent studies were also screened to identify additional potentially relevant citations that were not captured in the initial search results.

### Inclusion and exclusion criteria

The review aimed to include data available on field trials examining the effectiveness of SIT, IIT, or combined SIT-IIT for *Aedes* mosquitoes, emphasizing epidemiological outcomes. Consequently, review articles, protocols, case reports, opinion pieces, and predictive simulations were excluded from the analysis. Additionally, trials lacking a control group were excluded. Untranslated foreign language (not in English) articles were also excluded. During the second stage of assessment, studies focusing solely on mosquito surveillance without reporting human health outcomes or *Aedes*-borne disease data were not considered, to streamline the epidemiological findings.

### Quality assessment and data extraction

The quality of the included literature was assessed by two researchers working independently to ensure reliability. Initial screening, based on titles and abstracts, was followed by a comprehensive full-text review. Key information, which included study design, setting, duration, intervention details such as the mosquito species, sterilizing agent, and number of mosquitoes released, and outcomes, was extracted into pre-established templates. Methodological rigor, bias, confounding factors, data integrity, and statistical analyses were evaluated. Discrepancies were resolved through discussion and consensus. In the second phase, studies reporting epidemiological outcomes were evaluated in depth, with an emphasis on adherence to predefined inclusion criteria, such as alignment with the Population, Intervention, Comparator, Outcome (PICO) framework, and adherence to recognized reporting standards [19]. This rigorous assessment ensured the inclusion of studies that provided comprehensive data and met established methodological standards, thereby strengthening the validity of the review's conclusions.

### Data synthesis and gap analysis

Data synthesis involved a two-phase approach: initial extraction of key study information into standardized templates, followed by thematic analysis to identify patterns and trends. Studies were grouped by intervention type (SIT, IIT, and combined SIT-IIT), geographical region, and outcomes. For gap analysis, synthesized data were compared with existing knowledge to highlight methodological inconsistencies and under-researched areas. Special focus was given to epidemiological outcomes to assess the impact on human health and the methodological rigor was evaluated to identify ways of enhancing the design of future studies.

## Results

### Search results and overview of the trials

**Fig. 1:**
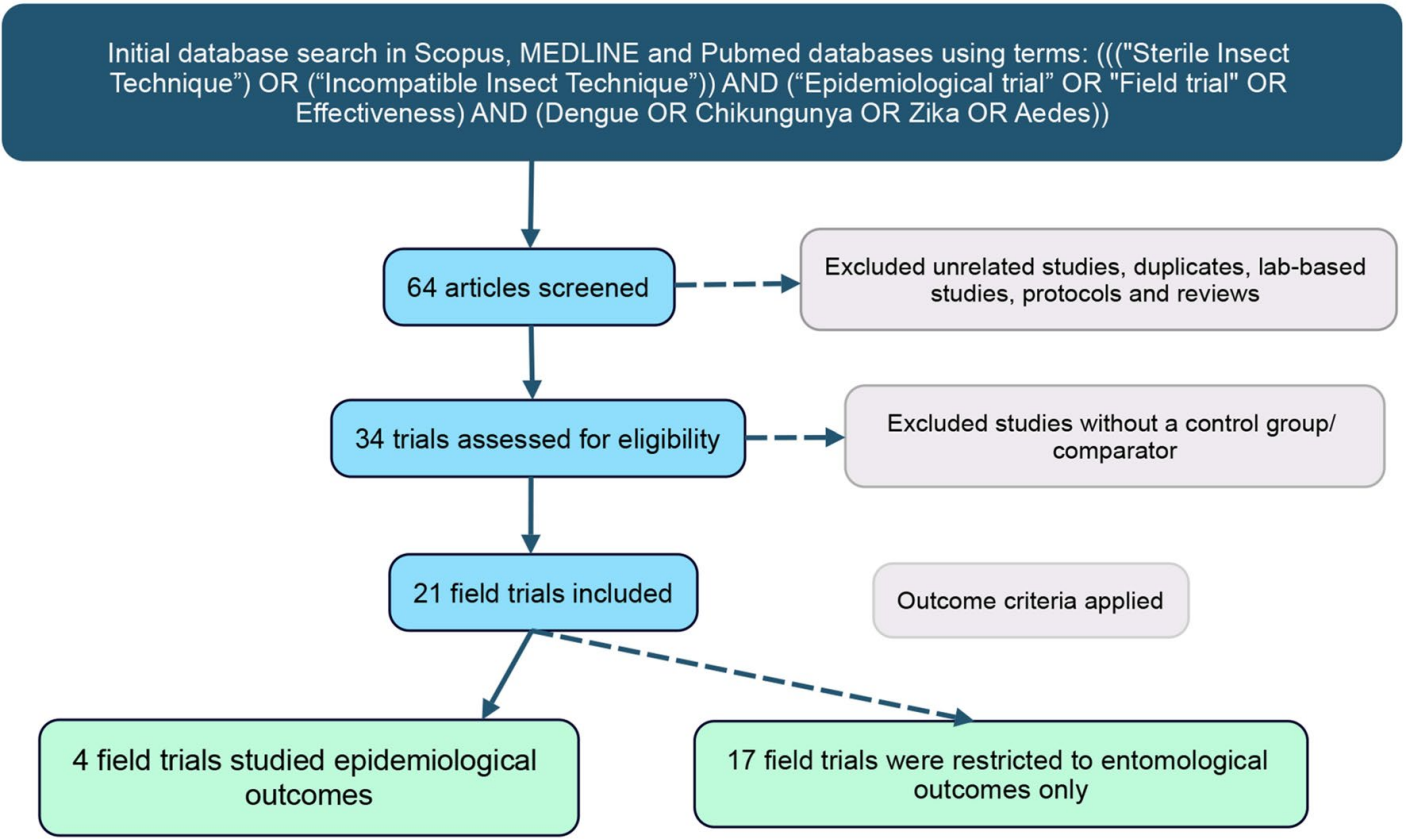
Flow diagram depicting the article selection for the review

The search yielded a total of 64 de-duplicated citations. Interestingly, the search also yielded a protocol for a randomized controlled trial investigating the efficacy of IIT-SIT for dengue control [20]. The full text of 34 trials in line with the selection criteria was evaluated and 13 trials among them were excluded considering the absence of a comparison group. The scope was narrowed to 21 field trials examining the effectiveness of SIT, IIT, or combined SIT-IIT for *Aedes* mosquitoes, as shown in Fig. 1. Only 4 field trials explicitly measured the epidemiological outcomes in terms of disease incidence and risk ratios. The full text of all the trials included in the review was available in English language. The results reflect the progress in trial methodologies published from 2012 to August 2024.

### Geographic distribution

**Fig. 2:**
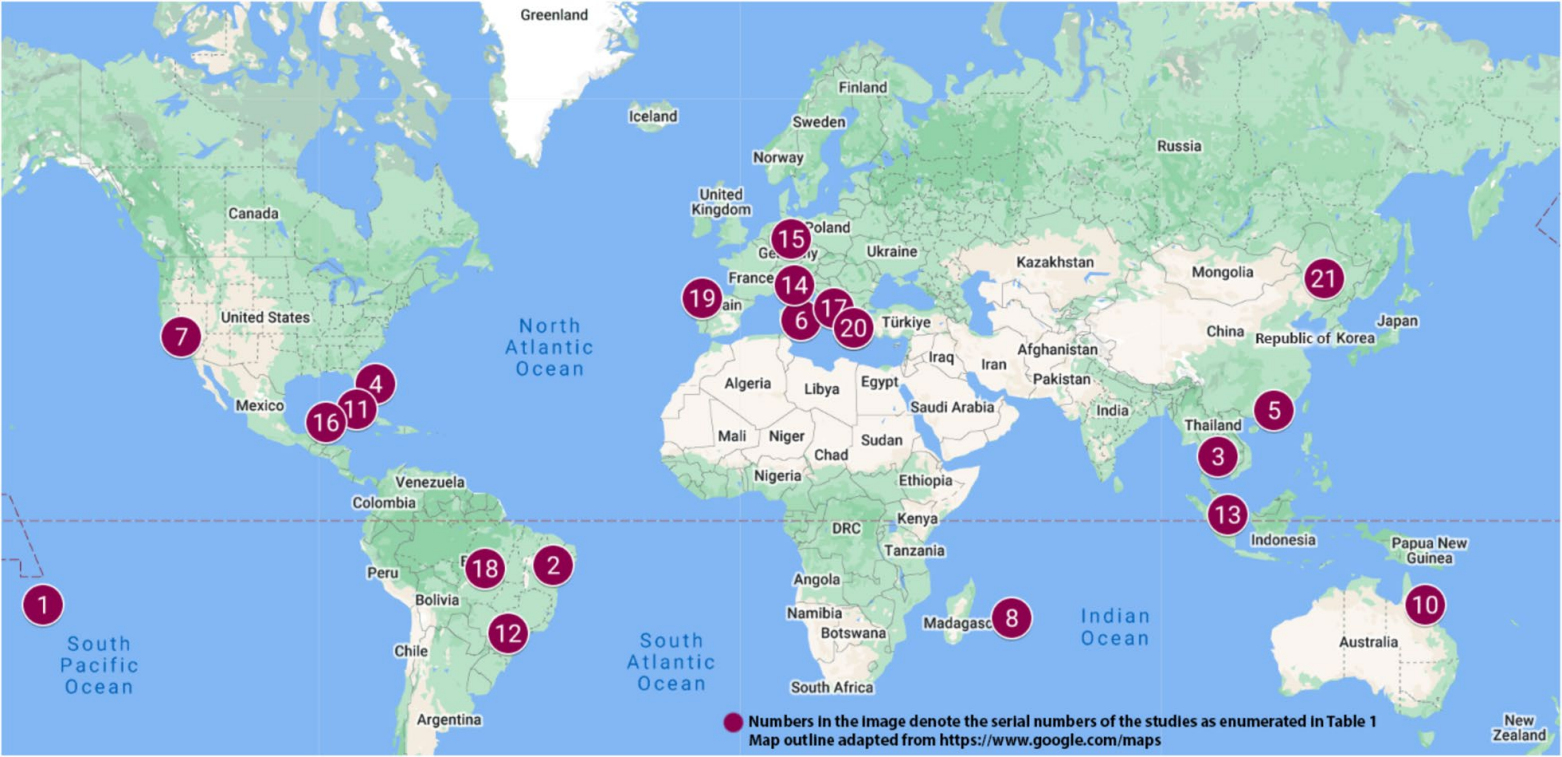
Geographical distribution of field trials employing sterile insect technique or incompatible insect technique for *Aedes* mosquito control

Several field trials have explored the use of SIT for *Aedes* mosquito control by releasing sterile males of *Aedes aegypti* or *Ae. albopictus* in various locations. The European Region and the Americas have led extensive trials, particularly in Brazil [21–23], Mexico [24], the United States [25, 26], Greece [27, 28] and Italy [29, 30]. Notable studies have also emerged from the Western Pacific Region, particularly in Singapore [31, 32], Australia [33] and China [15]. In contrast, the African, Eastern Mediterranean, and Southeast Asia Regions have seen fewer trials, with limited efforts in Indonesia and Thailand [34]. While Brazil, Mexico, Singapore, Indonesia, Thailand, and several Southeast Asian countries are endemic for dengue and experience regular outbreaks, significant epidemiological evaluations of the impact of the SIT/IIT have been conducted primarily in Brazil and Singapore. The geographic distribution of the SIT trials included in this review is illustrated in Fig. 2. Expanding SIT trials in underrepresented regions can provide critical data on its effectiveness and challenges across diverse ecological and sociopolitical settings, improving global strategies for *Aedes* control.

### Methodological advances in SIT and IIT trials for *Aedes* control

The trials have evolved building on previous successes in designing the intervention, methodology, implementation, and outcome assessment. These studies have explored different release techniques, tailored to the specific contexts. The strategies and outcomes are summarized in Table 1. Although several SIT-based field trials have been conducted for *Aedes* mosquito control, very few studies reported epidemiological outcomes. Notably, two trials employing SIT and two trials utilizing *Wolbachia*-based IIT reported epidemiological outcomes, such as a reduction in the incidence of dengue and the associated risk ratios (RR). These four trials are detailed in Table 2. Though all the studies followed a non-randomized design with comparators, they vary widely in the methodology. While Poncio et al. [22] attempted a cross-over technique to adjust for the baseline differences, Lim et al. [32] used synthetic controls to adjust and reweight the pre-intervention trends in a quasi-experimental framework [17, 18].

**Table 1:** Field trials on SIT/IIT/SIT with IIT with a control group against *Aedes* mosquito

Sl. No	References	Study design	Duration	Mosquito species used for SIT	Sex (male/female)	Sterilizing agent	Location	Ecological setting (spatial scale)	No. of mosquitoes released	No. of releases	Outcomes
1	O’Connor et al. (2012) [47]	Open release field trial with a control group	30 weeks	*Aedes aegypti*	Male	*Wolbachia* wMel	French Polynesia	Island	117,000	3	• *Ae. Aegypti* population suppression • Decrease in egg hatch with only three out of 25,000 eggs hatching
2	Carvalho et al. (2015) [21]	• Pilot trial • Mark, release, recapture with a control group	1 year	*Aedes aegypti*	Male	SIT using RIDL	Brazil	Islands (5.5 hectare)	185,000	18	• 95% *Ae*. *aegypti* population reduction based on adult trap data • 81% A*e*. *aegypti* population reduction based on ovitrap indices
3	Kittayapong et al. (2019) [34]	• Non randomised trial with a control group	6 months	*Aedes aegypti*	Male	Gamma-ray	Thailand	Semi-rural (0.65 km^2^)	437,980	24	• *Ae. aegypti* population suppression • 84% reduction in egg hatch rate (*OR* = 0.16) compared to 61.2% in the control area. The average suppression efficiency was 80.64 ± 9.36%
4	Mains et al. (2019) [25]	Mark release recapture experiments with an intervention and control group	5 months	*Aedes aegypti*	Male	Fluorescent dust	Florida, United States	Urban (~ 170 acres)	680,000	2	• *Ae. aegypti* population reduced in the WB1 male released area • A significant reduction in egg hatch (32–62%) was observed
5	Zheng et al. (2019) [15]	Field trial combining community participation with control group	10 months	*Aedes albopictus*	Male	*Wolbachia* wAlb	China	Islands (3.3 km^2^)	Site 1: 52.7 million in 2016 and 92.6 million in 2017 Site 2: 19.7 million in 2016 and 32.1 million in 2017	35	• *Ae. albopictus* population suppression • 62% population reduction yearly in the released site compared to the control site
6	Caputo et al. (2020) [29]	Open-release field trial with a control group	9 weeks	*Aedes albopictus*	Male	*Wolbachia* wPip	Central Rome, Italy	Urban (∼ 2.7 hectare)	26,680	6	• The average sterile-to-wild male ratio was 7:10 • 30% of females collected at the released spots were 100% sterile compared to control sites
7	Crawford et al. (2020) [26]	Non randomised field trial with a control group • Automated processes for large-scale suppression	26 weeks	*Aedes aegypti*	Male	*Wolbachia*	California, United States	Urban (293 hectare)	14,376,511	3	• *Ae. aegypti* population suppression • The female *Ae. aegypti* population was 95.5% lower in released areas compared to the control area
8	Iyaloo et al. (2020) [48]	Non-randomised field trial with a control group	1 year 2 months	*Aedes albopictus*	Male	Gamma-ray	Mauritius	Rural (3 hectare)	60,000	5	• *Ae. albopictus* population suppression • Sterile *Ae. albopictus* males could successfully compete with wild males
9	Balatsos et al. (2021) [27]	Non-randomised controlled trial	3 months	*Aedes albopictus*	Male	Gamma-ray	Greece	Urban (5 hectare)	111,000	7	• *Ae. albopictus* population suppression • A significant reduction in egg hatch rate from 14 to 54% was recorded in the SIT plot compared to the control plot
10	Beebe et al. (2021) [33]	Field trial with control group in three sites	25 weeks	*Aedes aegypti*	Male	*Wolbachia* wMel	Australia	Urban and sub-urban (Site 1—44 hectare; Site 2—65 hectare; Site 3—85.5 hectare)	56 million	69	• Above 80% *Ae. aegypti* population suppression
11	Gato et al. (2021) [36]	Non-randomised field trial with control group	5 months	*Aedes aegypti*	Male	Gamma-ray	Cuba	Sub-urban (50 hectare)	1,270,000	15	• No eggs were collected in the treatment area for up to 6 weeks, indicating suppression of the wild *Ae. aegypti* • Significant reduction in mean egg hate rate (79.77% egg hatch rate per week)
12	Poncio et al. (2021) [22]	Field trial with a cross-over design	1 year 4 months	*Aedes aegypti*	Male	Double-stranded RNA and Thiotepa (0.1–0.6%)	Brazil	Urban (50 hectare)	10,000,000 and 6,000,000	49	• 91.4% *Ae. aegypti* population reduction in released area
13	Project *Wolbachia*– Singapore Consortium (2021) [31]	Field trial with control group	46 weeks	*Aedes aegypti*	Male	*Wolbachia* wAlb along with a low dose X-ray	Singapore	Urban (4.39 hectare)	46,000	92	• *Ae. aegypti* population suppression • 91% and 66% reduction in the egg hatch rate • 71–88% lower dengue incidences compared to control sites
14	Balestrino et al. (2022) [30]	Non randomised field trial with a control group in three sites	2 weeks	*Aedes albopictus*	Male	Gamma-ray	Northern Italy	Rural (Site 1—14 hectare; Site 2—07 hectare; and Site 03—07 hectare)	48,000	2	• A higher survival rate of 80.0 ± 6.4% was observed • This trial confirmed the negligible effects of irradiation and marking procedures on the quality of the males released • The mean recapture rates were 0.27–1.7%
15	Becker et al. (2022) [49]	Field trial combining community participation, door-to-door control and SIT with a control group	6 months	*Aedes albopictus*	Male	Gamma-ray	Southern Germany	Urban (17 hectare)	320,000	18	• *Ae. albopictus* population suppression • Egg sterility was 84.7 ± 12.5% and 62.7 ± 25.8% in the released area, and 14.6 ± 7.3% in the control area
16	Martín-Park et al. (2022) [24]	Controlled before-and-after quasi-experimental field trial	6 months	*Aedes aegypti*	Male	*Wolbachia* wAlbB	Mexico	Sub-urban (50 hectare)	1,270,000	48	• 50.0–75.2% *Ae. aegypti* population reduction • Egg hatch rate significantly decreased by 76.5%
17	Velo et al. (2022) [50]	Non-randomised field trial with a control group	4 weeks	*Aedes albopictus*	Male	Gamma-ray	Albania	Urban (20 hectare)	48,011	2	• *Ae. albopictus* population suppression • The natural fertility in the untreated area was 98.24%, while it was 74.10% in the treated site
18	Poncio et al. (2023) [23]	Non-randomized controlled trial	2 years	*Aedes aegypti*	Male	Double-stranded RNA and thiotepa (0.6%)	Southern Brazil	Urban (200 sterile male mosquitoes per hectare)	59 million	84	• 98.7% *Ae. aegypti* population suppression • 97% lower dengue incidence compared to the control sites
19	Tur et al. (2023) [51]	Field trial with control group	2 years	*Aedes albopictus*	Male	Gamma-ray	Spain	Urban (45 hectare)	9,818,300	8	• *Ae. albopictus* population suppression • 70–80% reduction in the adult female and egg collection compared to the control area
20	Balatsos et al. (2024) [28]	Open-release field trial with a control group	22 weeks	*Aedes albopictus*	Male	Gamma-ray	Greece	Urban (40 hectare)	662,000	30	• *Ae. albopictus* population suppression • 78% reduction in egg density
21	Lim et al. (2024) [32]	Field trial with Synthetic control method in four sites	209 weeks	*Aedes aegypti*	Male	*Wolbachia* wMel	Singapore	Urban (Site 1—62.7 hectare; Site 2—114 hectare; Site 3—508.8 hectare; Site 4—347.3 hectare)	218.8 million	1–7 per week	• *Ae. aegypti* population suppression • 56.88% reduction in dengue incidence

*SIT* Sterile insect technique, *IIT* incompatible insect technique, *RIDL* release of insects carrying a dominant lethal, *RNA* ribonucleic acid, *OR* odds ratio

**Table 2:** Trials using SIT, IIT or SIT with IIT against *Aedes* mosquitoes reporting epidemiological outcomes

Studies	Study design	Site	Intervention strategy	Mosquitoes released and sample size	Accounting for bias	Epidemiological outcomes
Poncio et al. Brazil (2021) [22]	Field trial with a control site adopting a cross-over design (non-randomized)	Two comparable urban settings (São Pedro and Aeroporto) in Southern Brazil as intervention and control sites alternatively during the two intervention periods	Weekly release of sterile male mosquitoes using treatment that includes double-stranded RNA and thiotepa (0.1% to 0.6%) in two phases for 8 months and 5 months respectively	10 million mosquitoes were released in the first intervention period (INT1) and 6 million in the second (INT2) At risk population inhabiting the treated areas in São Pedro and Aeroporto was 4500 and 6000 respectively	Reversing of the intervention and control sites with a wash-out period of 5 months to account for baseline site-specific differences	• During INT1, the intervention site had 15.9 fold lower incidence of dengue compared to the control site [rate ratio (RR): 0.0627; (95% *CI*: 0.0414–0.0949)] • In INT2, the RR was 0.0737(95% *CI*: 0.0501–0.1083), showing a 13.7 times lower dengue incidence in the intervention group
Project *Wolbachia*– Singapore Consortium, 2021 [31]	Field trial with a control group (non-randomized)	Two high-rise housing estates in Singapore-Yishun and Tampines	Weekly release of *w*AlbB infected *Aedes aegypti* male mosquitoes using combined IIT with SIT for 15 weeks in Yishun and 31 weeks in Tampines	6–7 treated male mosquitoes were released per resident per week accounting for a total of 46,000 mosquitoes Initially covered 6175 households, later expanded to 13,510 households	Analysing the core release zones, buffer release zones, and non-release zones in both study settings to account for spillover effect	• The core and buffer release zones in the two intervention sites demonstrated significantly lower dengue incidences, ranging from 71% (95% *CI:* 43–87%) to 88% (95% *CI:* 57–99%) compared to control sites • Increasing distance from the cores correlated with higher dengue incidences in non-release areas, suggesting a positive spillover effect
Poncio et. al Brazil (2023) [23]	Field trial with a control group (non-randomized)	Ortigueira city served as the intervention site. One neighbouring city was used as a control for ovitrap collections, while four neighbouring cities acted as controls for epidemiological data analysis	Weekly release of mosquitoes treated with double-stranded RNA and thiotepa (0.6%) from November 2020 to July 2022	200 treated mosquitoes per hectare × number of viable larvae were released, accounting for a total of 59 million mosquitoes. 21,000 people resided in the intervention site	• Comparison of dengue incidence of neighboring cities with similar dengue epidemiological profiles • Additionally, pre and post-comparisons were also done	• The dengue incidence rate ratio (IRR) indicated an 89.1% reduction (95% *CI:* 95.5%–82.3%) in the intervention site compared to the control cities over the 2 years of intervention • The number of dengue cases decreased by up to 97.7% (95% *CI:* 95.7%–99.7%) throughout the intervention period, employing the monthly moving averages method
Lim et al. Singapore(2024) [32]	Large-scale field trial employing a quasi-experimental methodology with synthetic controls	Yishun and Tampines (large towns) implementing an expanding release strategy and Bukit Batok and Choa Chu Kang (smaller towns) opting a targeted-release approach	Twice weekly release of *w*AlbB-infected *Aedes aegypti* male mosquitoes produced using a combined IIT with SIT approach in 3 towns out of the 4 and high-fidelity sex sorting in the other town from epidemiological week (EW) 27, 2018, to EW 26, 2022	A total of 218.8 million mosquitoes were released, averaging 1–7 treated mosquitoes per study site resident per week	• Synthetic controls were used to adjust and reweight the pre-intervention trends in dengue incidence rates and covariates • Subgroup analysis done by age, sex, and dengue case type(clustered/sporadic) • Assessed the trend of intervention efficacies with coverage differences	• The intervention efficacy showed a concurrent increase with coverage. An aggregate efficacy of 56.90% (95% *CI*: 51.88–58.46%) was observed, despite incomplete coverage. The maximum efficacy reached was 77.28% • There were 2242 reported cases of dengue per 100,000 people in the intervention sites compared to 3941 cases in the control sites • Subgroup analysis revealed consistent intervention efficacy

*SIT* Sterile insect technique, *IIT* incompatible insect technique

Poncio et al. can be considered a preliminary approach to assess the epidemiological impact, by estimating the incidence through sourcing data from the passive epidemiological surveillance system of the health system in Brazil [22]. The same team expanded the study area to conduct another non-randomized controlled trial, with before and after comparisons, as well as comparisons with neighboring cities [23]. Though systematic entomological assessment was carried out in the intervention and control areas, the data on dengue incidence was sourced from the national disease surveillance system. This data included clinically suspected dengue cases and laboratory confirmation (PCR) only in severe/fatal cases, pregnant women, and children. Four neighboring cities with a comparable pattern of dengue incidence over the last two decades were selected as control areas. Though the study mentions the intervention to be accessible, reliable, scalable, and reproducible, the methodology did not seem to assess indicators of these outcomes. Lim et al. used virologically confirmed dengue cases reported through the Ministry of Health, Singapore to calculate the dengue incidence in the study areas [32]. Yearly intervention efficacy was analyzed alongside changes in the intervention coverage to assess the impact of different phases of dengue transmission. They also considered the possible confounding of age, gender, and transmission pattern (clustering) during analysis.

### Epidemiological outcomes

The Brazilian team [22, 23] conducted two trials on the SIT technique using double-stranded RNA and thiotepa-treated male *Ae. aegypti* mosquitoes [22, 23]. The initial trial (2021) achieved a 91.4% reduction in *Ae. aegypti* progeny (RR = 0.0627, 95% *CI:* 0.0414–0.0949) and observed a 15.9-fold decrease in the dengue incidence in phase 1 (8 months), followed by a 13.7-fold reduction in the 5 months post-cross-over (RR = 0.0737; 95% *CI:* 0.0501–0.1083) [22]. In the subsequent trial (2023), the scope was extended to the entire Ortigueira city in Brazil, where they released 59 million sterile male mosquitoes from November 2020 to July 2022. The team reported a remarkable 98.7% reduction in live progeny of *Ae. aegypti* mosquitoes, and an 89.1% (95% *CI:* 82.3–95.5%) reduction in the dengue incidence rate ratio in the intervention site compared to neighboring cities serving as epidemiological controls [23]. Furthermore, throughout the 2-year intervention period, there was a significant decline in the number of dengue cases with a maximum decrease of 97.7% (95% *CI:* 95.7–99.7%), as determined by the monthly moving averages method.

Project *Wolbachia*–Singapore Consortium team studied SIT–IIT-based X-ray sterilized *Ae. aegypti* males in two sites, namely Yishun and Tampines, Singapore [31]. The sterile male releases were conducted for 15 weeks in Yishun and 31 weeks in Tampines. A significant level of 91% and 66% reduction in the hatch rates of eggs was observed in two sites resulting in 71% (95% *CI:* 43–87%) to 88% (95% *CI:* 57–99%) lower dengue incidences in 2019 compared to control sites. Notably, this study also investigated changes in dengue incidence within buffer zones, revealing a positive spillover effect on both dengue incidence and *Ae. aegypti* population up to 1 km from intervention cores, highlighting an added benefit of IIT. Lim et al. studied the SIT–IIT activity of wAlbB-infected *Ae. aegypti* male mosquitoes in Yishun, Tampines, Bukit Batok, and Choa Chu Kang, Singapore [32]. This large-scale field trial released around 218.8 million mosquitoes demonstrating a concurrent increase in intervention efficacy with coverage. Across all towns and years, an aggregate efficacy of 56.88% (95% *CI:* 51.88–58.46%) was observed, despite an aggregate coverage of 34.5%, with the maximum efficacy reaching 77.28%. Moreover, the study revealed an aggregate intervention efficacy of 63.6% (95% *CI:* 61.04–66.00%) among clustered cases divided across 4 years, with the maximum reaching 82.9%. In terms of case aversion, the study reported averting a total of 516.9, 2114.6, 361.7, and 906.9 cases in 2019, 2020, 2021, and 2022, respectively. Additionally, this study demonstrated the replication of intervention effects across age groups, sexes, and dengue case types (clustered or sporadic) through subgroup analysis.

Although some studies have focused solely on entomological outcomes, efforts have been made to model the epidemiological impact. Carvalho et al. [21] applied the disease transmission threshold model, which was developed by Focks et al. [35] and is based on pupae per person, temperature, and seroprevalence. Using this model, they estimated that a reduction in pupae per person from 0.7 to 0.04 post-treatment observed in the study area, would be sufficient to prevent dengue epidemic transmission, even under adverse conditions.

### Considerations on cost and community engagement

Zheng et al. and Kittayapong et al. detailed the comprehensive community engagement strategies used before and during the open field releases [15, 34]. These strategies included stakeholder meetings, household visits, active health communication, public updates, and feedback channels, all aimed at building community understanding and support. Kittayapong et al. further report that 4.29% of households in the intervention area withdrew from the study, citing fear of mosquito bites as the reason [34]. Community engagement and communication strategies are also reported in several other studies [21, 24, 27, 33, 36], while they lack a systematic evaluation of community engagement through process and outcome indicators.

The cost of SIT and IIT remains competitive. The small pilot trial by Iyaloo et al. [37] estimated costs of EUR 582 per hectare per week for sterile male mosquito treatment, which was considerably higher than the EUR 54–216 per hectare per week reported in a similar Chinese pilot trial [15]. However, the specific breakdown of these costs was not detailed. Separately, Martín-Park et al. estimated a cost of EUR 385.28 for twice-weekly releases of 4000 *Ae. aegypti* males over a 50-hectare area, excluding the construction expenses of a mosquito-rearing facility but covering implementation costs [24]. This estimate did not account for the substantial costs associated with community engagement and surveillance. Although some studies have attempted to assess the costs of SIT and IIT techniques, the majority of the trials have not systematically evaluated the cost-effectiveness of these interventions using available entomological and epidemiological outcomes.

### Challenges

The authors have cited several challenges in the conduct of the trials. Poncio et al. [23] emphasized the importance of initiating interventions before the mosquito season while considering key bottlenecks, such as regulatory approvals, during the planning phase. They also highlighted the risk of mosquito population rebounds, noting that, *Ae. aegypti* eggs can remain dormant for over a year and human movement can lead to re-introductions of vector, necessitating robust entomological surveillance. Similarly, the Project *Wolbachia*–Singapore Consortium [31] reported a rapid rebound in the adult mosquito population and egg hatch rates post-intervention, which they attributed to the small site sizes. Their findings underscored the need for a very high threshold of wAlb mosquitoes to establish dominance over wild-type mosquitoes after intervention. However, they observed that once releases were conducted over larger areas with sufficient buffer zones, mosquito suppression could be maintained with lower release volumes, even when the IIT–SIT strain exhibited reduced fitness.

Additionally, technological and logistical challenges persist, including the high costs of mosquito production, storage, transport, and release, as well as public engagement and securing necessary authorizations and approvals [23, 27]. Crawford et al. noted that automation significantly improved the consistency of larval rearing, the precision of sex separation, and the accuracy of mosquito release, effectively addressing issues like low production yields, uncompetitive males, and high rates of female contamination [26].

## Discussion

The study explored and synthesized data from SIT, IIT, and combined SIT–IIT field trials against *Aedes* mosquitoes. The review provides a comprehensive overview of the interventions, study methodology, outcomes, and potential bias. Most studies focused on assessing entomological impacts, such as the mating competitiveness of sterile males, population suppression levels, and technical aspects of mosquito release strategies. Several trials evaluated optimal release rates and patterns to enhance coverage and mating success, while others analyzed the stability and persistence of sterile male populations in target areas. Notably, the majority of these trials reported significant reductions in mosquito populations, and four trials have shown positive epidemiological impact. In regions like Europe, where *Ae. aegypti* mosquitoes are present but arbovirus circulation or outbreaks are infrequent, the primary objective of SIT/IIT trials shifts from disease prevention to improving quality of life by mitigating the nuisance of mosquito bites [38]. In such contexts, these trials can significantly enhance public well-being while contributing to a broader understanding of vector control strategies. However, it is important to note that some trials conducted in locations with high endemicity or at risk of outbreaks of *Aedes*-borne arboviruses, such as in the Americas, Asia, and Africa, did not measure the effects of the intervention on disease transmission and burden. This may be due to challenges like financial and logistical constraints, as well as the need for enhanced surveillance systems to capture epidemiological outcomes more effectively. Many trials were also limited in spatial and temporal scope, often being pilot studies or conducted in restricted geographic areas and over short durations, which further constrained their ability to evaluate epidemiological impacts comprehensively. All the studies adopted a non-randomized controlled trial design, potentially limiting the level of evidence from a disease control program perspective. Notably, the upcoming multi-site trial by Ong et al. in Singapore will assess the efficacy of IIT–SIT in reducing dengue by combining randomized controlled trials (RCT) and test-negative design [20]. The study is a two-arm, non-blinded cluster-randomized trial in high-rise residential complexes in Singapore, aimed at assessing whether large-scale deployment of *Wolbachia-*infected male mosquitoes can significantly reduce dengue incidence. However, the study protocol excludes active dengue surveillance but additionally estimates the odds ratio of *Wolbachia* exposure distribution among confirmed dengue cases versus test-negative controls.

All the SIT/IIT trials published to date evaluating epidemiological outcomes have relied on national surveillance data to assess dengue incidence. Consequently, it's prudent to interpret the findings with caution, given that a significant portion of the illness persists sub-clinically and consequently goes unreported [39]. A recent systematic review suggests that 54% of dengue infections remain asymptomatic and clinically undetectable [40]. However, it’s worth noting that this limitation likely applies to control sites as well in comparative statistics. Enhancing the studies with active surveillance data or seroprevalence estimates, as discussed later, could improve their accuracy. The minutes of the meeting of the WHO Vector Control Advisory Group (VCAG) in 2023 report a planned study utilizing the SIT technique on two islands of French Polynesia—Tahiti and Tetiaroa. This study aims to assess the efficacy of SIT in reducing wild mosquito populations and dengue transmission through a structured release of sterile males. The study methodology incorporates active surveillance and serosurveys. However, VCAG notes that the non-randomized study design may limit the weight of the data obtained [41]. Further, the majority of studies reported in this review have overlooked coverage and implementation indicators. Notably, the study by the Project *Wolbachia*–Singapore Consortium in 2021 demonstrates a positive spillover effect through the analysis of buffer zones. While all studies mention ethical considerations and regulatory approvals, further insights into community engagement would greatly enhance their value.

As further investigations unfold in this domain, addressing certain fundamental methodological constraints in upcoming trials is critical. To demonstrate the public health effectiveness of new interventions, the WHO recommends conducting RCTs and cluster-randomized trials over at least two transmission seasons [16]. Further, the trials may utilize various other designs influenced by disease patterns, available resources, personnel, and logistical considerations like step wedge, cross-over or factorial design, and non-RCTs on a case-to-case basis. Measuring the epidemiological impact of SIT or IIT to reduce dengue transmission is complex. Contrary to individual-level interventions, these initiatives target entire populations within a given location or area targeted by sterile male releases. Hence, large-scale deployment is crucial to ensure the capture of adequate data on disease incidence for meaningful analysis [16, 42]. Further, there is a possibility of ‘noises’ and biases due to spillover and importation of sporadic cases which may complicate the analysis. Effective evaluation hinges on comparing outcomes in treated and control zones, necessitating the establishment of buffer zones to prevent interference. Mitigating the biases requires robust epidemiological designs accounting for geographic clustering, subject movement tracking, and other potential confounders. Establishing an optimal-sized longitudinal cohort to track seroconversion rates in children in the study population can aid in dealing with reporting biases by outlining data on the incidence and relative risk of dengue infection [42]. Additionally, the data can be enhanced by active surveillance of human infections by sampling the geographical clusters around dengue index cases [23, 24]. Blinding of human and mosquito samples before laboratory testing can be considered, given that true placebo treatment is not feasible in these studies. However, some argue that a cluster randomized controlled trial may not be the optimal approach, as it is essential to continuously learn and adapt mosquito release strategies and monitoring tools based on effectiveness and community factors during the study period, while also considering the significant costs involved [43].

Given the complexity of implementing the intervention and the pace of expansion, it is advisable to monitor the settings for at least 2 years to capture meaningful endpoint measures. This observation should include tracking key entomological indicators such as mosquito density, sterility rates, and vector competence, as well as epidemiological outcomes like disease incidence and seroconversion rates. Assessments of cost-effectiveness, feasibility, acceptability, and safety are equally important and should be evaluated alongside the entomological and epidemiological data.

While the literature acknowledges the technical feasibility of the intervention through large-scale pilot trials, safety concerns persist within communities [27, 28]. The fear of unintentionally releasing sub-sterile males or residual females, which could result in mosquito reproduction, continues to raise apprehension [44]. Additionally, the use of radiation or genetic modification to sterilize mosquitoes may evoke ecological concerns, despite research consistently showing minimal impact on ecosystems [16, 45]. Achieving a consensus between technical scientific evidence and social perceptions is paramount for the success of such interventions. Alphey et al. modeled cost estimates for employing sterile insect techniques, indicating approximately USD 2 to 30 per case averted [46]. Notably, this cost was considerably lower than the mean direct and indirect costs associated with the disease, which ranged from USD 86 to 190 per dengue case. Larger-scale operations might offer better cost-effectiveness due to economies of scale, where the average cost per unit of output decreases as the scale of the operation increases [15, 37, 46]. However, there remains a gap in the literature regarding trials that accurately assess the cost-effectiveness of sterile male mosquito release interventions. The lack of a comprehensive and systematic cost-effective analysis is a missed opportunity, particularly given the promising results in mosquito population suppression and disease management demonstrated in trials across various regions.

Future research should focus on improving methodological rigor through robust epidemiological designs that account for geographic clustering, subject movement, and confounders, alongside active surveillance and longitudinal cohort studies. The methodology should be meticulously designed using the appropriate and feasible approaches that take into account the disease patterns and resource availability of the specific setting. Expanding multi-country trials, incorporating systematic cost-effectiveness evaluations, and using standardized guidelines will enhance comparability and generalizability. Further studies should assess the scalability and sustainability by modeling long-term rebound effects and operational feasibility in both urban and rural settings. Community engagement, social acceptance, and ethical considerations are vital, making it necessary to conduct in-depth qualitative studies to align technical findings with public perceptions. Furthermore, trials should consider integrating SIT/IIT into existing vector-borne disease control ecosystems to enhance current strategies, such as source reduction, larviciding, adult mosquito control measures, and personal protection. This approach can assess the impact of adding SIT/IIT to integrated vector management strategies to better inform context-specific policies for controlling *Aedes*-borne diseases.

Our review has certain limitations. The search encompassed Scopus, MEDLINE, and PubMed databases and was limited to field trials published in the English language. Additionally, grey literature, ongoing clinical trials, and reviews about the topic were not incorporated into our analysis. There also exists the potential for publication bias in this review, as studies demonstrating successful reductions in mosquito populations are more likely to be reported and disseminated. Consequently, the absence of negative or inconclusive studies may skew the overall interpretation of the effectiveness of these interventions. However, we have tried to mitigate these limitations by employing a comprehensive search strategy that includes preprints and by incorporating a snowball search method to identify additional relevant studies.

## Conclusions

The review highlights promising strides in SIT and IIT to complement and enhance *Aedes* mosquito control, alongside a noticeable gap in trials evaluating the epidemiological outcomes within this domain. While notable successes have been observed, including substantial reductions in *Aedes* populations and dengue incidence rates in the intervention areas, challenges persist in standardizing methodologies and accurately assessing epidemiological impacts. The diversity in study designs underscores the complexity of evaluating population-based interventions and emphasizes the need for rigorous epidemiological frameworks. Addressing gaps in surveillance data, enhancing community engagement, and considering cost-effectiveness, safety, and acceptability elements are essential for the successful implementation of SIT and IIT strategies. Researchers in this field can leverage the WHO guidelines and support of WHO’s Vector Control Advisory Group to design and implement trials that yield robust evidence on the real-world effectiveness of these interventions in combatting *Aedes*-borne diseases.

## Supplementary Information

Additional file 1. Databases and search strategies.

## Data Availability

The datasets analysed in the current study are available from the corresponding author on reasonable request.

## Abbreviations

SIT: Sterile insect technique
IIT: Incompatible insect technique
WHO: World Health Organization
DALY: Disability adjusted life years
PRISMA-ScR: Preferred Reporting Items for Systematic Reviews and Meta-Analyses Extension for Scoping Reviews

## References

[1] WHO. WHO scales up response to worldwide surge in dengue. https://www.who.int/news-room/feature-stories/detail/who-scales-up-response-to-worldwide-surge-in-dengue. Accessed 25 Mar 2024.

[2] WHO. Dengue and severe dengue. https://www.who.int/news-room/fact-sheets/detail/dengue-and-severe-dengue. Accessed 25 Mar 2024.

[3] European Centre for Disease Prevention and Control Dengue cases January-December 2023; 2024. https://www.ecdc.europa.eu/en/publications-data/dengue-cases-january-december-2023. Accessed 14 Oct 2024.

[4] WHO. Dengue: WHO Health Emergency Appeal 2024. https://www.who.int/publications/m/item/dengue-who-health-emergency-appeal-2024. Accessed 26 Jun 2024.

[5] Girard M, Nelson CB, Picot V, Gubler DJ. Arboviruses: a global public health threat. Vaccine. 2020;38:3989–94. 32336601 10.1016/j.vaccine.2020.04.011 PMC7180381

[6] Puntasecca CJ, King CH, LaBeaud AD. Measuring the global burden of chikungunya and Zika viruses: a systematic review. PLoS Negl Trop Dis. 2021;15:e0009055. 33661908 10.1371/journal.pntd.0009055 PMC7932082

[7] Kolimenakis A, Heinz S, Wilson ML, Winkler V, Yakob L, Michaelakis A, et al. The role of urbanisation in the spread of *Aedes* mosquitoes and the diseases they transmit—a systematic review. PLOS Negl Trop Dis. 2021;15: e0009631. 34499653 10.1371/journal.pntd.0009631 PMC8428665

[8] Kaura T, Walter NS, Kaur U, Sehgal R, Kaura T, Walter NS, et al. Different strategies for mosquito control: Challenges and alternatives. In: mosquito research—recent advances in pathogen interactions, immunity, and vector control strategies. Intech Open; 2022. 10.5772/intechopen.104594.

[9] Roiz D, Wilson AL, Scott TW, Fonseca DM, Jourdain F, Müller P, et al. Integrated *Aedes* management for the control of *Aedes*-borne diseases. PLoS Negl Trop Dis. 2018;12:e0006845. 30521524 10.1371/journal.pntd.0006845 PMC6283470

[10] Huang YJS, Higgs S, Vanlandingham DL. Biological control strategies for mosquito vectors of arboviruses. Insects. 2017;8:21. 28208639 10.3390/insects8010021 PMC5371949

[11] Hendrichs J, Dyck AA, Robinson AS. Sterile insect technique: principles and practice in area-wide integrated pest management. Dordrecht: Springer; 2005.

[12] Dyck VA, Hendrichs J, Robinson AS, editors. Sterile insect technique: principles and practice in area-wide integrated pest management. 2nd ed. Boca Raton: CRC Press; 2021.

[13] Flores HA, O’Neill SL. Controlling vector-borne diseases by releasing modified mosquitoes. Nat Rev Microbiol. 2018;16:508–18. 29777177 10.1038/s41579-018-0025-0 PMC7612058

[14] Werren JH, Baldo L, Clark ME. Wolbachia: master manipulators of invertebrate biology. Nat Rev Microbiol. 2008;6:741–51. 18794912 10.1038/nrmicro1969

[15] Zheng X, Zhang D, Li Y, Yang C, Wu Y, Liang X, et al. Incompatible and sterile insect techniques combined eliminate mosquitoes. Nature. 2019;572:56–61. 31316207 10.1038/s41586-019-1407-9

[16] WHO. Guidance framework for testing the sterile insect technique as a vector control tool against *Aedes*-borne diseases. https://www.who.int/publications-detail-redirect/9789240002371. Accessed 1 May 2024.

[17] WHO. How to design vector control efficacy trials: guidance on phase III vector control field trial design provided by the Vector Control Advisory Group; 2017. https://iris.who.int/handle/10665/259688

[18] Tricco AC, Lillie E, Zarin W, O’Brien KK, Colquhoun H, Levac D, et al. PRISMA Extension for Scoping Reviews (PRISMA-ScR): Checklist and Explanation. Ann Intern Med. 2018;169:467–73. 30178033 10.7326/M18-0850

[19] Reeves BC, Gaus W. Guidelines for reporting non-randomised studies. Forsch Komplementarmed Klass Naturheilkd. 2004;11(Suppl 1):46–52. 15353903 10.1159/000080576

[20] Ong J, Ho SH, Soh SXH, Wong Y, Ng Y, Vasquez K, et al. Assessing the efficacy of male *Wolbachia*-infected mosquito deployments to reduce dengue incidence in Singapore: Study protocol for a cluster-randomized controlled trial. Trials. 2022;23:1023. 36528590 10.1186/s13063-022-06976-5 PMC9758775

[21] Carvalho DO, McKemey AR, Garziera L, Lacroix R, Donnelly CA, Alphey L, et al. Suppression of a field population of *Aedes aegypti* in Brazil by sustained release of transgenic male mosquitoes. PLoS Negl Trop Dis. 2015;9:e0003864. 26135160 10.1371/journal.pntd.0003864 PMC4489809

[22] Poncio LDC, dos Anjos FA, de Oliveira DA, Rebechi D, de Oliveira RN, Chitolina RF, et al. Novel sterile insect technology program results in suppression of a field mosquito population and subsequently to reduced incidence of dengue. J Infect Dis. 2021;224:1005–14. 33507265 10.1093/infdis/jiab049

[23] Poncio LDC, dos Anjos FA, de Oliveira DA, de Rosa AO, Silva BP, Rebechi D, et al. Prevention of a dengue outbreak via the large-scale deployment of sterile insect technology in a Brazilian city: a prospective study. Lancet Reg Health Am. 2023;21:100498. 37187486 10.1016/j.lana.2023.100498 PMC10176055

[24] Martín-Park A, Che-Mendoza A, Contreras-Perera Y, Pérez-Carrillo S, Puerta-Guardo H, Villegas-Chim J, et al. Pilot trial using mass field-releases of sterile males produced with the incompatible and sterile insect techniques as part of integrated *Aedes aegypti* control in Mexico. PLoS Negl Trop Dis. 2022;16:e0010324. 35471983 10.1371/journal.pntd.0010324 PMC9041844

[25] Mains JW, Kelly PH, Dobson KL, Petrie WD, Dobson SL. Localized control of *Aedes aegypti* (Diptera: Culicidae) in Miami, FL, via inundative releases of *Wolbachia*-infected male mosquitoes. J Med Entomol. 2019;56:1296–303. 31008514 10.1093/jme/tjz051

[26] Crawford JE, Clarke DW, Criswell V, Desnoyer M, Cornel D, Deegan B, et al. Efficient production of male *Wolbachia*-infected *Aedes aegypti* mosquitoes enables large-scale suppression of wild populations. Nat Biotechnol. 2020;38:482–92. 32265562 10.1038/s41587-020-0471-x

[27] Balatsos G, Puggioli A, Karras V, Lytra I, Mastronikolos G, Carrieri M, et al. Reduction in egg fertility of *Aedes albopictus* mosquitoes in Greece following releases of imported sterile males. Insects. 2021;12:110. 33513716 10.3390/insects12020110 PMC7911890

[28] Balatsos G, Karras V, Puggioli A, Balestrino F, Bellini R, Papachristos DP, et al. Sterile insect technique (SIT) field trial targeting the suppression of *Aedes albopictus* in Greece. Parasite. 2024;31:17. 38530210 10.1051/parasite/2024020 PMC10964849

[29] Caputo B, Moretti R, Manica M, Serini P, Lampazzi E, Bonanni M, et al. A bacterium against the tiger: preliminary evidence of fertility reduction after release of *Aedes albopictus* males with manipulated *Wolbachia* infection in an Italian urban area. Pest Manag Sci. 2020;76:1324–32. 31603613 10.1002/ps.5643

[30] Balestrino F, Puggioli A, Malfacini M, Albieri A, Carrieri M, Bouyer J, et al. Field performance assessment of irradiated *Aedes albopictus* males through mark–release–recapture rials with multiple release points. Front Bioeng Biotechnol. 2022;10: 876677. 35928955 10.3389/fbioe.2022.876677 PMC9344911

[31] Consortium TPW-S, Ching NL. *Wolbachia*-mediated sterility suppresses *Aedes aegypti* populations in the urban tropics. 2021. 10.1101/2021.06.16.21257922.

[32] Lim JT, Bansal S, Chong CS, Dickens B, Ng Y, Deng L, et al. Efficacy of *Wolbachia*-mediated sterility to reduce the incidence of dengue: a synthetic control study in Singapore. Lancet Microbe. 2024;5(5):e422–32. 38342109 10.1016/S2666-5247(23)00397-X

[33] Beebe NW, Pagendam D, Trewin BJ, Boomer A, Bradford M, Ford A, et al. Releasing incompatible males drives strong suppression across populations of wild and *Wolbachia*-carrying *Aedes aegypti* in Australia. PNAS. 2021;118(41): e2106828118. 34607949 10.1073/pnas.2106828118 PMC8521666

[34] Kittayapong P, Ninphanomchai S, Limohpasmanee W, Chansang C, Chansang U, Mongkalangoon P. Combined sterile insect technique and incompatible insect technique: the first proof-of-concept to suppress *Aedes aegypti* vector populations in semi-rural settings in Thailand. PLOS Negl Trop Dis. 2019;13: e0007771. 31658265 10.1371/journal.pntd.0007771 PMC6837763

[35] Focks DA, Brenner RJ, Hayes J, Daniels E. Transmission thresholds for dengue in terms of *Aedes aegypti* pupae per person with discussion of their utility in source reduction efforts. Am J Trop Med Hyg. 2000;62:11–8. 10761719

[36] Gato R, Menéndez Z, Prieto E, Argilés R, Rodríguez M, Baldoquín W, et al. Sterile insect technique: Successful suppression of an *Aedes aegypti* field population in Cuba. Insects. 2021;12:469. 34070177 10.3390/insects12050469 PMC8158475

[37] Iyaloo DP, Bouyer J, Facknath S, Bheecarry A. Pilot suppression trial of Aedes albopictus mosquitoes through an integrated vector management strategy including the sterile insect technique in Mauritius. bioRxiv. 2020. 10.1101/2020.09.06.284968.

[38] Olson MF, Juarez JG, Kraemer MUG, Messina JP, Hamer GL. Global patterns of aegyptism without arbovirus. PLoS Negl Trop Dis. 2021;15:e0009397. 33951038 10.1371/journal.pntd.0009397 PMC8128236

[39] WHO. Dengue- Global situation. https://www.who.int/emergencies/disease-outbreak-news/item/2023-DON498. Accessed 1 May 2024.

[40] De Santis O, Bouscaren N, Flahault A. Asymptomatic dengue infection rate: a systematic literature review. Heliyon. 2023;9: e20069. 37809992 10.1016/j.heliyon.2023.e20069 PMC10559824

[41] WHO. Nineteenth meeting of the WHO Vector Control Advisory Group. https://www.who.int/publications/i/item/9789240087699. Accessed 14 Oct 2024.

[42] Wolbers M, Kleinschmidt I, Simmons CP, Donnelly CA. Considerations in the design of clinical trials to test novel entomological approaches to dengue control. PLoS Negl Trop Dis. 2012;6:e1937. 23209869 10.1371/journal.pntd.0001937 PMC3510076

[43] Lambrechts L, Ferguson NM, Harris E, Holmes EC, McGraw EA, O’Neill SL, et al. Assessing the epidemiological impact of *Wolbachia* deployment for dengue control. Lancet Infect Dis. 2015;15:862–6. 26051887 10.1016/S1473-3099(15)00091-2 PMC4824166

[44] Jiménez-Alejo A, Pacheco-Soriano AL, Liedo P, Marina CF, Bond JG, Rodríguez-Ramos JC, et al. Acceptance of a sterile male releases pilot project to reduce *Aedes aegypti* (Linnaeus, 1762) (Diptera: Culicidae) populations and its associated factors: a community-based cross-sectional survey in South Chiapas, Mexico. Acta Trop. 2022;233:106573. 35768038 10.1016/j.actatropica.2022.106573

[45] Bellini R. Safety, regulatory and environmental issues with sterile insect technique-based mosquito vector control in European countries. Rev Sci Tech. 2022;41(1):170–7. 35925624 10.20506/rst.41.1.3314

[46] Alphey N, Alphey L, Bonsall MB. A model framework to estimate impact and cost of genetics-based sterile insect methods for dengue vector control. PLoS ONE. 2011;6: e25384. 21998654 10.1371/journal.pone.0025384 PMC3187769

[47] O’Connor L, Plichart C, Sang AC, Brelsfoard CL, Bossin HC, Dobson SL. Open release of male mosquitoes infected with a *Wolbachia* biopesticide: field performance and infection containment. PLoS Negl Trop Dis. 2012;6:e1797. 23166845 10.1371/journal.pntd.0001797 PMC3499408

[48] Iyaloo DP, Facknath S, Bheecarry A. Investigating the effects of low temperature and compaction on the quality of adult radio-sterilised *Aedes albopictus* (Diptera: Culicidae) males in view of their optimal transport to the pilot sterile release site in Mauritius. Int J Trop Insect Sci. 2020;40:53–62.

[49] Becker N, Langentepe-Kong SM, Tokatlian Rodriguez A, Oo TT, Reichle D, Lühken R, et al. Integrated control of *Aedes albopictus* in Southwest Germany supported by the sterile insect technique. Parasit Vectors. 2022;15(1):9. 34983608 10.1186/s13071-021-05112-7 PMC8727083

[50] Velo E, Balestrino F, Kadriaj P, Carvalho DO, Dicko A, Bellini R, et al. A mark-release-recapture study to estimate field performance of imported radio-sterilized male *Aedes albopictus* in Albania. Front Bioeng Biotechnol. 2022;10: 833698. 36051578 10.3389/fbioe.2022.833698 PMC9424856

[51] Tur C, Almenar D, Zacarés M, Benlloch-Navarro S, Pla I, Dalmau V. Suppression trial through an integrated vector management of *Aedes albopictus* (Skuse) based on the sterile insect technique in a non-isolated area in Spain. Insects. 2023;14:688. 37623398 10.3390/insects14080688 PMC10455479

