# Supplementary material for: Innovative sterile male release strategies for Aedes mosquito control: progress and challenges in integrating evidence of mosquito population suppression with epidemiological impact
Source: Infect Dis Poverty. 2024 Dec 3;13:91. doi: 10.1186/s40249-024-01258-5 (PMC11613880; doi:10.1186/s40249-024-01258-5)
Supplement: Supplementary file 1 — Additional file 1. Databases and search strategies. [file 40249_2024_1258_MOESM1_ESM.docx]

**PubMed Search Query**

**Basic search**

**(("Sterile Insect Technique” OR “Incompatible Insect Technique”) AND ("Field trial" OR Effectiveness) AND (Dengue OR Chikungunya OR Zika OR Aedes))**

1. ("Sterile Insect Technique"[Title/Abstract] OR "Incompatible Insect Technique"[Title/Abstract]) AND ("Field trial"[Title/Abstract] OR "Effectiveness"[Title/Abstract]) AND ("Dengue"[Title/Abstract] OR "Chikungunya"[Title/Abstract] OR "Zika"[Title/Abstract] OR "Aedes"[Title/Abstract])
2. (("Sterile Insect Technique"[Title/Abstract] OR "Incompatible Insect Technique"[Title/Abstract]) AND ("Field trial"[Title/Abstract] OR "Effectiveness"[Title/Abstract]) AND ("Dengue"[Title/Abstract] OR "Chikungunya"[Title/Abstract] OR "Zika"[Title/Abstract] OR "Aedes"[Title/Abstract])) AND ((ffrft[Filter]) AND (fha[Filter]) AND (fft[Filter]) AND (english[Filter]))

**Modifications**

1. (("Sterile Insect Technique” OR “Incompatible Insect Technique” OR Sterile male release) AND ("Field trial" OR pilot trial OR intervention study OR controlled trial OR Effectiveness OR community trial) AND (Dengue OR Chikungunya OR Zika OR Aedes))
2. (("Sterile Insect Technique"[All Fields] OR "Incompatible Insect Technique"[All Fields]) OR ("infertility"[MeSH Terms] OR "infertility"[All Fields] OR "sterile"[All Fields] OR "sterility"[All Fields] OR "sterilant"[All Fields] OR "sterilants"[All Fields] OR "sterilisation"[All Fields] OR "sterilization, reproductive"[MeSH Terms] OR "sterilization"[All Fields] OR "reproductive"[All Fields]) OR "reproductive sterilization"[All Fields] OR "sterilization"[All Fields] OR "sterilization"[MeSH Terms] OR "sterilisations"[All Fields] OR "sterilised"[All Fields] OR "steriliser"[All Fields] OR "sterilisers"[All Fields] OR "sterilising"[All Fields] OR "sterilise"[All Fields] OR "sterilize"[All Fields] OR "sterilizations"[All Fields] OR "sterilized"[All Fields] OR "sterilizer"[All Fields] OR "sterilizers"[All Fields] OR "sterilizes"[All Fields] OR "sterilizing"[All Fields]) AND ("male"[MeSH Terms] OR "male"[All Fields]) AND "release"[All Fields] OR "released"[All Fields] OR "releases"[All Fields] OR "releasing"[All Fields])) AND ("Field trial"[All Fields] OR "pilot"[All Fields] OR "piloted"[All Fields] OR "piloting"[All Fields] OR "pilot"[MeSH Terms] OR "pilot"[All Fields]) AND ("clinical trials as topic"[MeSH Terms] OR ("clinical"[All Fields] AND "trials"[All Fields] AND "topic"[All Fields]) OR "clinical trials as topic"[All Fields] OR "trial"[All Fields] OR "trial s"[All Fields] OR "trials"[All Fields])) OR ("clinical trial"[Publication Type] OR "clinical trials as topic"[MeSH Terms] OR "intervention study"[All Fields]) OR ("controlled"[All Fields] AND ("clinical trials as topic"[MeSH Terms]) OR ("effect"[All Fields] OR "effecting"[All Fields] OR "effective"[All Fields] OR "effectiveness"[All Fields] OR "effectivity"[All Fields] OR "effects"[All Fields]) OR (("community"[All Fields] OR "community’s"[All Fields] OR "communities"[All Fields] OR "community"[All Fields]) AND ("clinical trials as topic"[MeSH Terms]) AND ("dengue"[MeSH Terms] OR "dengue"[All Fields] OR ("chikungunya fever"[MeSH Terms] OR ("chikungunya"[All Fields] AND "fever"[All Fields]) OR "chikungunya fever"[All Fields] OR "chikungunya"[All Fields]) OR ("zika virus"[MeSH Terms] OR ("zika"[All Fields] AND "virus"[All Fields]) OR "zika virus"[All Fields] OR "zika"[All Fields] OR "zika virus infection"[MeSH Terms] OR ("zika"[All Fields] AND "virus"[All Fields] AND "infection"[All Fields]) OR "zika virus infection"[All Fields]) OR ("aedes"[MeSH Terms] OR "aedes"[All Fields]))) AND ((ffrft[Filter]) AND (fha[Filter]) AND (fft[Filter]) AND (english[Filter]))
3. (("Sterile Insect Technique"[Title/Abstract] OR "Incompatible Insect Technique"[Title/Abstract] OR "sterile male release"[Title/Abstract]) AND ("Field trial"[Title/Abstract] OR "pilot trial"[Title/Abstract] OR "intervention study"[Title/Abstract] OR "controlled trial"[Title/Abstract] OR "Effectiveness"[Title/Abstract] OR "community trial"[Title/Abstract]) AND ("Dengue"[Title/Abstract] OR "Chikungunya"[Title/Abstract] OR "Zika"[Title/Abstract] OR "Aedes"[Title/Abstract])) AND ((ffrft[Filter]) AND (fha[Filter]) AND (fft[Filter]) AND (english[Filter]))

**Scopus Search Query**

1. TITLE-ABS-KEY((( "Sterile Insect Technique" OR "Incompatible Insect Technique") AND ("Field trial"  OR effectiveness) AND (dengue OR chikungunya OR zika OR Aedes))) AND (LIMIT-TO ( LANGUAGE, "English"))
2. TITLE-ABS-KEY ((("Sterile Insect Technique" OR "Incompatible Insect Technique" OR sterile AND male AND release ) AND ("Field trial" OR pilot AND trial OR intervention AND study OR controlled AND trial OR effectiveness OR community AND trial) AND (dengue OR chikungunya OR zika OR Aedes))) AND (LIMIT-TO (LANGUAGE, "English"))

**Web of Science Search Query**

1. TITLE-ABS-KEY ((("Sterile Insect Technique" OR "Incompatible Insect Technique" OR sterile AND male AND release) AND ("Field trial" OR pilot AND trial OR intervention AND study OR controlled AND trial OR effectiveness OR community AND trial) AND (dengue OR chikungunya OR zika OR Aedes))) AND (LIMIT-TO (LANGUAGE, "English"))
2. TITLE-ABS-KEY (("Sterile Insect Technique” OR “Incompatible Insect Technique” OR Sterile male release) AND ("Field trial" OR pilot trial OR intervention study OR controlled trial OR Effectiveness OR community trial) AND (Dengue OR Chikungunya OR Zika OR Aedes)) (Topic) and English (Languages)
